# Diospyrobezoar (Persimmon Bezoar)-Induced Intestinal Obstruction in an Older Patient: A Case Report

**DOI:** 10.7759/cureus.87850

**Published:** 2025-07-13

**Authors:** Masato Habuka, Moeri Yamagiwa, Masataka Yonezawa, Asa Ogawa, Suguru Yamamoto

**Affiliations:** 1 Division of Nephrology, Niigata Prefectural Shibata Hospital, Shibata, JPN; 2 Division of Clinical Nephrology and Rheumatology, Niigata University Graduate School of Medical and Dental Sciences, Niigata City, JPN

**Keywords:** bezoar, chronic kidney disease, diospyrobezoar, intestinal obstruction, older patient, persimmon, phytobezoar

## Abstract

Diospyrobezoar is a bezoar caused by excessive persimmon (*Diospyros kaki*) consumption, typically occurring in individuals with risk factors such as a history of gastric surgery, diabetes, or advanced age. We report the case of a 93-year-old man who presented with anorexia, nausea, and vomiting. Computed tomography revealed a bezoar-induced obstruction in the proximal jejunum. Despite initial management with Coca-Cola (The Coca-Cola Company, Atlanta, Georgia, United States) lavage and endoscopic fragmentation, surgical intervention was required because of persistent symptoms and intestinal ulceration. A 5 × 5-cm mass composed of >98% tannin was removed, confirming the presence of a diospyrobezoar. Further history assessment revealed that the patient consumed two persimmons daily. The patient recovered uneventfully after surgery and was discharged with dietary guidance. The present case highlights the importance of identifying diospyrobezoar-induced intestinal obstruction in older persimmon consumers, even in the absence of common risk factors, such as prior gastric surgery or diabetes. Early diagnosis based on imaging findings and dietary history, followed by timely surgical treatment, is essential to prevent the development of severe complications. Moreover, educating patients about the risks of excessive persimmon consumption is essential to prevent this condition.

## Introduction

Bezoars are masses of indigestible material that accumulate in the gastrointestinal tract, most often in the stomach. Among them, diospyrobezoars are a distinct subtype formed from excessive consumption of persimmons (*Diospyros kaki*), which are rich in soluble tannins and dietary fibers [1]. When exposed to gastric acid, these tannins can polymerize and interact with proteins and cellulose to form a hard, cohesive mass that resists digestion [2].

Diospyrobezoars are relatively rare but can cause significant clinical symptoms, particularly when they lead to gastrointestinal obstruction. Typically, they are associated with several risk factors, including prior gastric surgery, diabetes, and advanced age. These risk factors impair mechanical digestion and transit, creating a favorable environment for bezoar formation [2]. However, cases of intestinal obstruction in older adults without these common risk factors are extremely rare. We present the case of a 93-year-old man who developed intestinal obstruction caused by a diospyrobezoar, despite having no history of gastric surgery or diabetes.

## Case presentation

A 93-year-old man with stage 5 chronic kidney disease (CKD) of unknown etiology was referred to our hospital with a two-day history of anorexia, nausea, and vomiting. He had previously opted for conservative kidney management because of his advanced age; however, he had not received dietary counseling regarding the consumption of potassium-rich fruits. He had a history of hypertension and benign prostatic hyperplasia but had no history of gastric surgery or diabetes. His medications included sacubitril/valsartan, amlodipine, carvedilol, daprodustat, and tamsulosin.

On admission, his vital signs were as follows: temperature, 37.2°C; blood pressure, 174/66 mmHg; pulse, 77 beats/minute; respiratory rate, 18 breaths/minute; and oxygen saturation, 100% on room air. He appeared ill and fatigued. Physical examination revealed abdominal tenderness without rebound, decreased bowel sounds, and bilateral pitting edema. Laboratory tests revealed anemia, renal dysfunction, and metabolic acidosis. Table 1 shows the clinical data of the patient.

**Table 1:** Laboratory test results at hospital admission ALP, alkaline phosphatase; ALT, alanine aminotransferase; AST, aspartate aminotransferase; BUN, blood urea nitrogen; Ca, calcium; CK, creatine kinase; Cl, Chloride; Cr, creatinine; CRP, c-reactive protein; K, Potassium; LDH, lactate dehydrogenase; Na, sodium; P, phosphorus; γ-GTP, gamma-glutamyl transpeptidase; T-Bil, total bilirubin; TP, total protein

Variable	Patient Value	Reference Range
Blood count		
White blood cells, /µL	9,700	3,300-8,600
Hemoglobin, g/dL	10.3	13.7-16.0
Platelets ×10^4^/µL	21.1	15.8-34.0
Blood Gas Analysis		
pH	7.3	7.35-7.40
Bicarbonate, mEq/L	20.0	21.0-28.0
Anion gap, mEq/L	17.4	10.0-20.0
Serum Chemistry		
TP, g/dL	7.4	6.6-8.1
Albumin, g/dL	3.2	4.1-5.1
BUN, mg/dL	93	8-20
Cr, mg/dL	4.48	0.65-1.0
Na, mEq/L	141	138-145
K, mEq/L	4.1	3.6-4.8
Cl, mEq/L	107	101-108
Ca, mg/dL	7.9	8-8.10.1
P, mg/dL	5.9	2.7-4.6
AST, U/L	19	13-30
ALT, U/L	8	10-42
γGTP, U/L	12	13-64
ALP, U/L	106	38-133
LDH, U/L	332	124-222
T-Bil, mg/dL	0.4	0.4-1.5
CK, U/L	276	59-248
CRP, mg/dL	1.13	0.00-0.1

**Figure 1:**
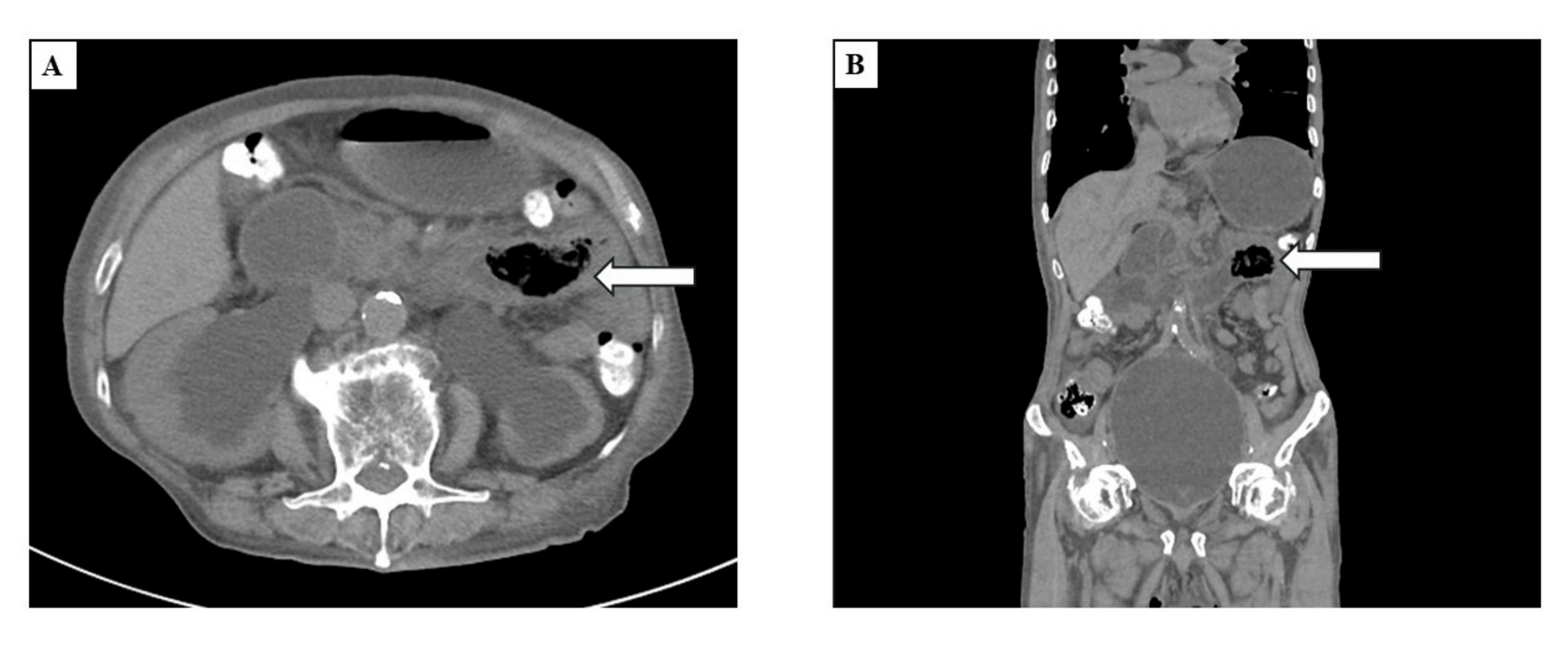
Computed tomography (A: axial view; B: coronal view) showing bilateral hydronephrosis and a massive gas-containing structure in the lumen of the proximal jejunum (arrow)

Abdominal computed tomography (CT) revealed bilateral hydronephrosis due to prostatic enlargement and a large gas-containing mass in the proximal jejunum, indicating a bezoar-induced intestinal obstruction (Figure 1).

**Figure 2:**
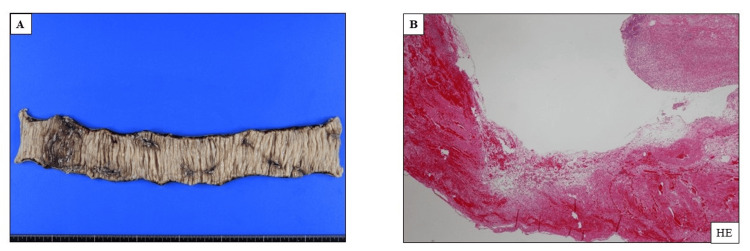
Histological findings of the small intestine samples obtained during laparotomy (A) Macroscopic examination showing multiple ulcers in the resected small intestine; (B) Hematoxylin & eosin staining showing an ulcer, neutrophil infiltration, and hemorrhage

A transnasal ileus tube was inserted to achieve proximal decompression, which reduced the intestinal dilation; however, it did not resolve the obstruction. Coca-Cola (The Coca-Cola Company, Atlanta, Georgia, United States) lavage was administered every 12 hours, and endoscopic fragmentation was attempted on day 6 of hospitalization. However, the removal of the bezoar was unsuccessful. Because of the ulceration of the adjacent intestinal wall, laparoscopic partial small bowel resection was performed on day 7. Histopathological examination of the resected specimen revealed multiple ulcers (Figure 2).

**Figure 3:**
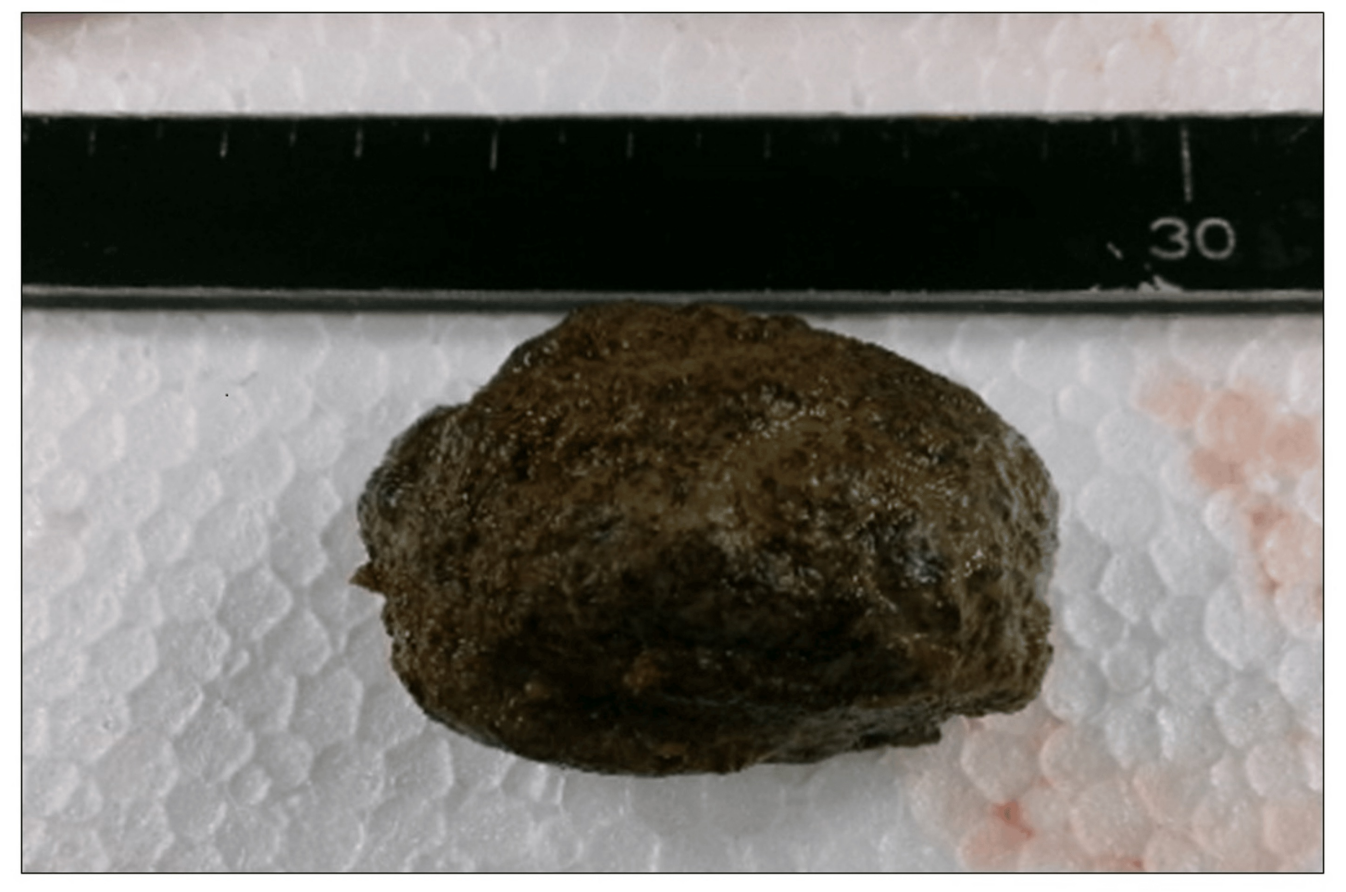
Image of the removed diospyrobezoar The diospyrobezoar was hard and rough, with a dark brown surface

A 5 × 5-cm bezoar was extracted (Figure 3), which contained over 98% tannin on component analysis, confirming it to be a diospyrobezoar.

Further, the patient’s dietary history revealed that he had a persimmon tree in his garden, and he consumed two persimmons daily. The patient’s postoperative course was uneventful, and he was discharged with instructions to limit his persimmon intake to only one fruit per day.

## Discussion

We reported a rare case of diospyrobezoar-induced intestinal obstruction requiring surgical resection in an older patient with no history of gastric surgery or diabetes.

Diospyrobezoars are formed when unripe persimmons, which contain high tannin concentrations, are ingested in large quantities. The tannins in the fruit form a coagulum that can cause diospyrobezoars [2]. Previous gastric surgery, presence of diabetes, and advanced age are the risk factors for diospyrobezoar formation. This condition results in gastric acidity, stasis, and impaired gastric motility [3]. Endoscopic examination and CT, which reveal the presence of an intraluminal mass with a mottled gas appearance and a dilated small bowel proximal to the obstruction, along with a careful review of the patient’s medical history, can help in the diagnosis of diospyrobezoar. In our case, although the patient had no history of surgery or diabetes, his clinical symptoms and imaging findings led to the suspicion of a bezoar; thus, he was diagnosed with a diospyrobezoar based on his dietary history and the results of the component analysis.

Intestinal obstruction caused by diospyrobezoars is rare and often requires urgent surgical intervention. A literature search using the terms “diospyrobezoar” or “persimmon bezoar” and “intestinal obstruction” or “ileus” revealed only five reported cases (Table 2) [4-8].

**Table 2:** Reported cases of diospyrobezoar-induced intestinal obstruction

Variables	Funamizu et al. (2015) [4]	Ha et al. (2007) [5]	de Toledo et al. (2012) [6]	Ohya et al. (2023) [7]	Zheng et al. (2014) [8]	Present Case
Age (years), sex	67, F	73, M	66, M	93, F	27, M	93, M
Duration of excessive persimmon ingestion	Regular intake of persimmon	Not mentioned	6 days	Regular intake of persimmon	2 weeks	Regular intake of persimmon
Underlying disease	Previous surgery for esophageal cyst and cesarean section	Hypereosinophilic syndrome and prednisolone use; previous Coca-Cola lavage for gastric diospyrobezoar	Diabetes mellitus; previous gastrectomy and vagotomy	Dementia; previous gastrectomy and cholecystectomy	Not mentioned	Hypertension; prostatic hypertrophy
Clinical symptoms	Abdominal distention and pain; Vomiting	Vomiting after 1 month of Coca-Cola lavage	Abdominal pain with signs of peritoneal irritation; Nausea	Nausea; Anorexia	Abdominal distention and pain; Nausea	Anorexia; Nausea; Vomiting
Diagnostic procedures	Radiograph CT	Radiograph Ultrasound	Radiograph	CT Gastroenterography Laparoscopy	Radiograph CT Endoscopy	CT Endoscopy
Treatment	Ileus tube Laparotomy	Laparotomy	Laparotomy	Ileus tube Laparotomy	Ileus tube; Coca-Cola lavage; Endoscopic fragmentation	Ileus tube; Coca-Cola lavage; Endoscopic fragmentation; Laparotomy
Diospyrobezoar size	7 cm × 4 cm	Size not mentioned; Three diospyrobezoar pieces were identified	5 cm × 12 cm	4 cm × 5 cm	5 cm × 6 cm	5 cm × 5 cm
Diospyrobezoar component analysis	Yes	Yes	Not mentioned	Yes	Not mentioned	Yes
Outcome	Recovery	Recovery	Recovery	Recovery	Recovery	Recovery
Prevention of recurrence	Not mentioned	Not mentioned	Dietary orientation	Not mentioned	Dietary orientation	Dietary orientation

All five cases were characterized by abdominal pain, nausea, and vomiting, which are typical symptoms of intestinal obstruction, and three of them had a history of upper gastrointestinal surgery. Although treatments such as Coca-Cola lavage and endoscopic fragmentation are available, diospyrobezoars are often resistant to dissolution because of their harder consistency, compared with other types of bezoars [9]. Thus, surgical excision is inevitable in patients with an intestinal obstruction or a refractory diospyrobezoar. Among the five reported cases, four required surgery (Table 2). Our patient exhibited ulceration of the surrounding intestinal tract, and further delays in surgical treatment could have resulted in fatal complications, including bacterial peritonitis. Diospyrobezoar-induced intestinal obstruction should be diagnosed early based on the patient’s medical history, typical symptoms, and imaging findings, and surgical intervention should be selected to minimize the mortality risk.

Our case is notable because it represents one of the few instances of diospyrobezoar-induced obstruction in an older patient with no prior gastric surgery or diabetes. In the 1980s, when persimmons became popular in Israel, the number of diospyrobezoar cases increased. However, the incidence considerably decreased after the public was warned about the risks associated with the overconsumption of persimmons [10], indicating that public education about such risks can reduce the incidence of this rare condition. Older adults are more prone to diospyrobezoar-induced obstruction because of decreased gastric motility and altered digestive function [11]. Therefore, clinicians should be aware of the risks of diospyrobezoar-induced obstruction in older individuals who consume persimmon. Moreover, educating patients about the risks of excessive persimmon consumption is essential for preventing this condition.

## Conclusions

The present case highlights the importance of identifying diospyrobezoar-induced intestinal obstruction in older individuals who consume persimmon, even in the absence of common risk factors, such as prior gastric surgery or diabetes. Early diagnosis based on the imaging findings and dietary history, followed by timely surgical treatment, is essential to prevent severe complications. Moreover, educating patients about the risks of excessive persimmon consumption is essential to prevent this condition.
